# Acoustic Eigenvalues of a Quasispherical Resonator: Second Order Shape Perturbation Theory for Arbitrary Modes

**DOI:** 10.6028/jres.112.013

**Published:** 2007-06-01

**Authors:** James B. Mehl

**Affiliations:** National Institute of Standards and Technology, Gaithersburg, MD 20899; 36 Zunuqua Trail, P.O. Box 307, Orcas, WA 98280

**Keywords:** acoustic cavity resonators, acoustic eigenvalues, quasispheres, shape perturbation theory

## Abstract

The boundary-shape formalism of Morse and Ingard is applied to the acoustic modes of a deformed spherical resonator (quasisphere) with rigid boundaries. For boundary shapes described by *r* = *a* [1 − *ε* ℱ(*θ*, *ϕ*)], where *ε* is a small scale parameter and ℱ is a function of order unity, the frequency perturbation is calculated to order *ε*^2^. The formal results apply to acoustic modes whose angular dependence is designated by the indices *ℓ* and *m*. Specific examples are worked out for the radial (*ℓ* = 0) and triplet (*ℓ* = 1) modes, for prolate and oblate spheroids, and for triaxial ellipsoids. The exact eigenvalues for the spheroids, and eigenvalue determined with finite-element calculations, are shown to agree with perturbation theory through terms of order *ε*^2^. This work is an extension of the author’s previous papers on the acoustic eigenfrequencies of deformed spherical resonators, which were limited to the second-order perturbation for radial modes [J. Acoust. Soc. Am. **71**, 1109-1113 (1982)] and the first order-perturbation for arbitrary modes [J. Acoust. Soc. Am. **79**, 278–285 (1986)].

## 1. Introduction

Spherical acoustic resonators [1] have been successfully applied to measurement of the universal gas constant [2] and to gas thermometry [3–7]. The radial (0, *n*) acoustic modes are well-suited for high-accuracy work because they are non-degenerate, well separated, insensitive to visco-thermal boundary effects, and only weakly dependent on the details of small shape imperfections [1,8,9]. Nearly-spherical resonators, now referred to as *quasispherical* resonators [10,11], have been designed to facilitate the measurement of the electromagnetic resonances for determination of the mean radius of the quasisphere. The speed of sound can be determined from the combination of measured acoustic and electromagnetic resonance frequencies [12]. The acoustic and electromagnetic eigenvalues for quasispherical resonators must be evaluated using approximation methods. In this paper, boundary-shape perturbation theory [13] is used to calculate the acoustic eigenvalues.

An idealized rigid spherical resonator of radius *a* has acoustic modes with the acoustic pressure proportional to the eigenfunctions

$$\Phi_{\ell nm}=j_{\ell }\left(k_{\ell n}r\right){ϒ}_{\ell m}(\theta ,\phi ),$$ (1)

where *j_ℓ_* (*ξ*) is a spherical Bessel function and ϒ*_ℓm_* is a linear combination of spherical harmonics

$${ϒ}_{\ell m}=\sum_{\mu =-\ell }^{\ell }\alpha_{m\mu }Y_{\ell \mu }$$ (2)

with coefficients *α_mµ_* chosen to make the ϒ*_ℓm_* real. The eigenvalues for the perfect spherical geometry are *k_ℓn_* = *ξ_ℓn_*/*a*, where *ξ_ℓn_* is the *n*th root of *j*′*_ℓ_* (*ξ_ℓn_*) = 0.

The bounding surface of a quasispherical resonator has the form

$$r_{s}=a[1-\varepsilon ℱ(\theta ,\phi )],$$ (3)

where *ε* is a scale parameter satisfying 0 < *ε* << 1 and *F* is a smooth, non-negative function of *θ* and *ϕ*. The *ℓ* = 0 eigenvalues of a quasisphere differ from those of a perfect sphere of the same volume by a fraction of order *ε*^2^ or higher [1, 8]. The non-radial acoustic modes of a perfect sphere occur in rnultiplets of degeneracy 2*ℓ* + 1. Typically, this degeneracy is split to order *ε* in a quasisphere, but the mean eigenvalue of any multiplet differs from the corresponding eigenvalue of a perfect sphere of the same volume by an amount of order *ε*^2^ or higher [9]. The same is true of the electric and magnetic modes of a quasisphere with perfectly conducting walls [12].

In principle, a highly accurate measurement of the speed of sound in a gas can be made by measuring the acoustic and electromagnetic resonance frequencies of the same quasisphere. Geometric contributions to the error will then be of order *ε*^2^. If the shape is known, higher accuracy can be obtained if the theoretical coefficients of the *ε*^2^ perturbation terms can be calculated. This has already been achieved for the radial acoustic modes [8]. A corresponding theory for the electromagnetic modes, a subject of current research by the author, is much more complex, and is closely related to the theory of the second order shape perturbation theory for the non-radial acoustic modes, as developed in this paper. The results derived here will be useful in experimental studies of quasispheres which will compare the effects of shape on the acoustic and electromagnetic spectra. Also, the results enable the use of the lowest-frequency acoustic modes, the 11*m* triplet, to be used for high-accuracy work.

## 2. Formalism

**Fig. 1:**
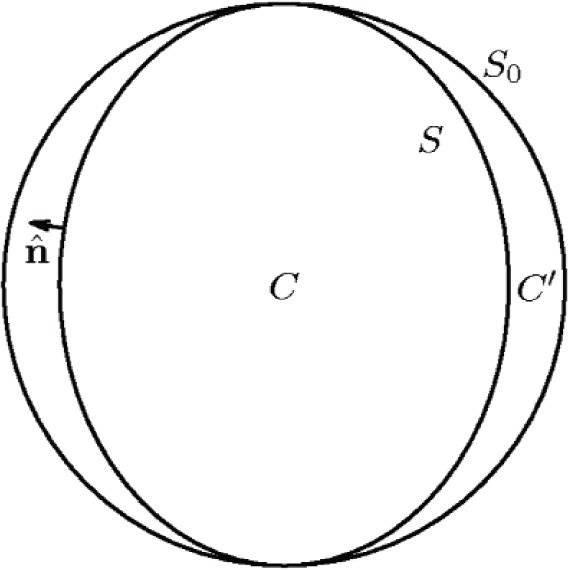
Perturbed cavity *C* (boundary *S*) within unperturbed cavity *C*_0_ (boundary *S*_0_). The region between *C* and *C*_0_ is designated *C*′.

Morse and Feshbach [13] (MF hereafter) present a formalism for calculating the eigenfrequencies of an acoustic cavity resonator *C* enclosed within an unperturbed cavity *C*_0_. Figure 1 illustrates the geometry; the surfaces enclosing *C* and *C*_0_ are designated *S* and *S*_0_, respectively, and the region between *C* and *C*_0_ is designated *C*′. The unperturbed cavity has a set of eigenfunctions Φ*_N_* and eigenvalues $k_{N}^{2}$, satisfying the Helmholtz equation

$$\left(\nabla^{2}+k_{N}^{2}\right)\Phi_{N}=0$$ (4)

in *C*_0_ and the Neumann boundary condition

$$\frac{\partial \Phi_{N}}{\partial n}\equiv \hat{n}\cdot \nabla \Phi_{N}=0$$ (5)

on *S*_0_. (For brevity, the subscript *N* in these equations, and other subscripts in capital letters, are used to represent sets of lower-case mode indices.)

The perturbed problem is defined by a surface *S* enclosed within *S*_0_, and enclosing a cavity *C*. The perturbed problem satisfies

$$\left(\nabla^{2}+k^{2}\right)\Phi =0$$ (6)

in *C* and the Neumann boundary condition

$$\frac{\partial \Phi }{\partial n}=0$$ (7)

on *S*. A second-order expression for the perturbed eigenvalue *k*^2^ is [MF Eq. (9.2.53)]

$$\begin{array}{l} \left(k^{2}-k_{N}^{2}\right)N_{NN}+\\ \sum_{P\neq N}\frac{\left[A_{NP}-\left(k^{2}-k_{N}^{2}\right)N_{NP}\right]\left[A_{PN}-(k^{2}-k_{P}^{2})N_{PN}\right]}{N_{PP}(k^{2}-k_{P}^{2})-A_{PP}}, \end{array}$$ (8)

where

$$A_{QP}=\int_{S}\Phi_{P}\frac{\partial \Phi_{Q}}{\partial n}dS$$ (9)

and

$$N_{PQ}=N_{QP}=\int_{C}\Phi_{P}\Phi_{Q}dV.$$ (10)

A more useful computational form for the integrals (9) can be obtained by applying the divergence theorem to Φ*_P_*∇Φ*_Q_* ± Φ*_Q_*∇Φ_P_ in *C* to obtain

$$A_{QP}+A_{PQ}=-\int_{C\prime }\left[2\nabla \Phi_{P}\cdot \nabla \Phi_{Q}-\left(k_{P}^{2}+k_{Q}^{2}\right)\Phi_{P}\Phi_{Q}\right]dV$$ (11)

and

$$A_{QP}-A_{PQ}=\left(k_{Q}^{2}-k_{P}^{2}\right)\int_{C\prime }\Phi_{P}\Phi_{Q}dV.$$ (12)

A negative sign occurs in these expressions because the outward normal from *C*′ on *S* is $-\hat{n}$. For Eq. (12), use was made of the the orthogonality of the unperturbed functions in *C*_0_ for *p* ≠ *q*:

$$\int_{C}\Phi_{P}\Phi_{Q}dV+\int_{C\prime }\Phi_{P}\Phi_{Q}dV=0,\;Q\neq P.$$ (13)

An expression for the diagonal terms follows directly from Eq. (11):

$$A_{PP}=\int_{C\prime }\left[k_{p}^{2}{\left|\Phi_{P}\right|}^{2}-{\left|\nabla \Phi_{P}\right|}^{2}\right]dV.$$ (14)

This is an integral over the region between *S* and *S*_0_ of a quantity proportional to the difference between the potential energy and the kinetic energy. The corresponding term for the perturbation of the electromagnetic modes has the same form [12]. An expression for the off-diagonal terms can be obtained from the sum of Eqs. (11) and (12):

$$A_{PQ}=\int_{C\prime }\left[k_{P}^{2}\Phi_{P}\Phi_{Q}-\nabla \Phi_{P}\cdot \nabla \Phi_{Q}\right]dV.$$ (15)

## 3. Deformed Spherical Resonator

Consider a deformed spherical resonator with a boundary surface *S* defined by Eq. (3).

When applied to a quasisphere with *S* defined by Eq. (3), the volume *C′*, and accordingly the integrals in Eqs. (14) and (15) are of order *ε*. Equation (8) can then be solved iteratively to obtain

$$\begin{array}{l} \frac{k^{2}-k_{N}^{2}}{k_{N}^{2}}=\frac{A_{NN}}{k_{N}^{2}N_{NN}}+\\ \;\sum_{P\neq N}\frac{{\left|A_{NP}\right|}^{2}}{N_{NN}N_{PP}k_{N}^{2}\left(k_{N}^{2}+k_{P}^{2}\right)}+O\left(\varepsilon^{3}\right). \end{array}$$ (16)

To evaluate Eq. (16) to order *ε*^2^, the numerator of the first term on the right must be calculated to *O*(*ε*^2^), the denominator to *O*(*ε*), the coefficients *A_NP_* in the sum term to *O*(*ε*). The normalization constants in the denominator of the sum term need only be calculated to *O*(1).

The acoustic modes of a perfect spherical resonator occur in multiplets with (2*ℓ* + 1)-fold degeneracy. Only the *ℓ* = 0 radial modes are nondegenerate. When calculating the perturbation series for nonradial modes, the coefficients *α_mµ_* in Eq. (2) should be chosen to make the coefficients *A_PQ_* zero for the modes with *k_P_* = *k_Q_*. Equation (12) shows that *A_NP_* = *A_PN_* exactly for degenerate pairs, so the proper choice of coefficients *α_mµ_* can be obtained by diagonalizing the submatrix [*A_NP_*] linking the multiplet terms.

More precisely, the off-diagonal terms of this sub-matrix must be of order *ε*^2^. Consider the application of Eq. (8) to the multiplet components. The first-order perturbation shift of each component is of order *ε*. In an iterative solution of Eq. (8) the order of the terms in the denominator of the sum terms would be *N_PP_* = *O*(1), $k^{2}-k_{N}^{2}=O(\varepsilon )$, and *A_PP_* = *O*(*ε*). The numerator is the square of $A_{NP}-\left(k^{2}-k_{N}^{2}\right)N_{PN}$. Both $k^{2}-k_{N}^{2}$ and $N_{NP}$ are of order *ε*, so if *A_NP_* = *O*(*ε*^2^) the numerator will be of order *ε*^4^, and the entire sum term of order *ε*^3^.

To get the first term in Eq. (16) to *O*(*ε*^2^), the numerator must be calculated to *O*(*ε*^2^), and the denominator to *O*(*ε*). The thickness of the integration volume *C*′ in Eq. (14) is of order *ε*, so the integrand of the numerator is needed to *O*(*ε*). Within *C*′ the radial derivative of the spherical Bessel function is of order *ε* so the function itself satisfies

$$j_{\ell }\left(k_{\ell n}r\right)=j_{\ell }\left(\xi_{\ell n}\right)+O\left(\varepsilon^{2}\right).$$ (17)

Integrals $N_{PQ}$ with *P* ≠ *Q* do not appear in Eq. (16), only normalization integrals for which the repeated indices are superfluous. The notation can hence be simplified by using an ordinary math font for N and a set of lower-case indices to designate the mode. In the new notation, the normalization integral in the denominator is

$$N_{\ell nm}=\int d\Omega \int_{0}^{r_{s}}{\left[j_{\ell }\left(k_{\ell n}r\right)\right]}^{2}{\left|{ϒ}_{\ell m}\right|}^{2}r^{2}dr,$$ (18)

which can be evaluated as the difference between an integral from *r* = 0 to *a* and an integral from *r_S_* to *a* to obtain, for *ℓnm* ≠ 010,

$$\begin{array}{l} N_{\ell nm}=\frac{a^{3}}{2}j_{\ell }{\left[\left(\xi_{\ell n}\right)\right]}^{2}\left[1-\frac{\ell (\ell +1)}{\xi_{\ell n}^{2}}\right]\\ \;-\varepsilon a^{3}{\left[j_{\ell }\left(\xi_{\ell n}\right)\right]}^{2}\int ℱ{\left|{ϒ}_{\ell m}\right|}^{2}d\Omega +O\left(\varepsilon^{2}\right). \end{array}$$ (19)

For the special case *nℓm* = 010, the eigenfunction is *j*_0_(*k*_01_*r*) = 1 and the eigenvalue is *ξ*_01_ = 0. The normalization constant is

$$N_{010}=\frac{a^{3}}{3}+O(\varepsilon ),$$ (20)

which differs from Eq. (19) by a factor of 2/3.

The function ℱ may itself depend on the scaling parameter *ε*; it is useful to make this explicit:

$$ℱ={ℱ}_{0}+\varepsilon {ℱ}_{1}+O\left(\varepsilon^{2}\right).$$ (21)

The first term in Eq. (16) then has the form

$$\frac{A_{\ell nm\ell nm}}{k_{\ell n}^{2}N_{\ell nm}}=2\varepsilon \frac{\int \left[\xi_{\ell n}^{2}{ϒ}_{\ell m}^{2}\left({ℱ}_{0}+\varepsilon {ℱ}_{1}-\varepsilon {ℱ}_{0}^{2}\right)-{\left|r\nabla {ϒ}_{\ell m}\right|}^{2}\left({ℱ}_{0}+\varepsilon {ℱ}_{1}\right)\right]d\Omega }{\xi_{\ell n}^{2}-\ell (\ell +1)-2\varepsilon \xi_{\ell n}^{2}\int {ℱ}_{0}{\left|{ϒ}_{\ell m}\right|}^{2}d\Omega }+O(\varepsilon^{3}).$$ (22)

(Note that, the operator *r*∇ appearing in this expression involves only angular derivatives.) The coefficients (15) in the perturbation series are only needed to *O*(*ε*), so the integrand is only needed to *O*(1); only the leading order of Eq. (18) is needed in the denominator. The sum in Eq. (16) simplifies to

$$\begin{array}{l} \sum_{\ell \prime n\prime m\prime \neq \ell nm}\frac{{\left|A_{\ell nm\ell \prime n\prime m\prime }\right|}^{2}}{N_{\ell nm}N_{\ell \prime n\prime m\prime }k_{\ell n}^{2}\left(k_{\ell n}^{2}-k_{\ell \prime n\prime }^{2}\right)}\\ \;=\sum_{\ell \prime n\prime m\prime \neq \ell nm}\frac{4\varepsilon^{2}{\left|B_{\ell m\ell \prime m\prime }^{\left(n\right)}\right|}^{2}}{\xi_{\ell n}^{2}-\ell (\ell +1)}\frac{\xi_{\ell \prime n\prime }^{2}}{\left[\xi_{\ell \prime n\prime }^{2}-\ell \prime (\ell \prime +1)\right]\left(\xi_{\ell n}^{2}-\xi_{\ell \prime n\prime }^{2}\right)} \end{array}$$ (23)

where

$$B_{\ell m\ell \prime m\prime }^{\left(n\right)}=\int \left[\xi_{\ell n}^{2}{ϒ}_{\ell \prime m\prime }{ϒ}_{\ell m}-r^{2}\nabla {ϒ}_{\ell \prime m\prime }\cdot \nabla {ϒ}_{\ell m}\right]{ℱ}_{0}d\Omega .$$ (24)

The sum over *n′* in Eq. (23) is

$$S_{\ell n\ell \prime }=\sum_{v=1}^{\infty }{}^{\prime }\frac{1}{\xi_{\ell n}^{2}-\xi_{\ell \prime v}^{2}}\frac{\xi_{\ell \prime v^{\prime }}^{2}}{\xi_{\ell \prime n}^{2}-\ell \prime (\ell \prime +1)},$$ (25)

where the prime on the summation symbol indicates the omission of the terms with *ℓ*′*ν* = *ℓn*. The sums are evaluated analytically in the Appendix using the technique of Ref [8]. The results for *ℓ*′ ≠ 0 are

$$S_{\ell n\ell \prime }=\{\begin{array}{ll} -\frac{j_{\ell \prime }\left(\xi_{\ell n}\right)}{2\xi_{\ell n}j_{\ell \prime }^{\prime }\left(\xi_{\ell n}\right)}, & \text{for}\;\ell \prime \neq \ell ,\\ \frac{\xi_{\ell n}^{2}-3\ell (\ell +1)}{4{\left[\xi_{\ell n}^{2}-\ell (\ell +1)\right]}^{2}}, & \text{for}\;\ell \prime =\ell , \end{array}$$ (26)

and, for *ℓ*′ = 0,

$$S_{\ell n0}=\{\begin{array}{ll} -\frac{1}{2\xi_{\ell n}^{2}}+\frac{j_{0}\left(\xi_{\ell n}\right)}{2\xi_{\ell n}j_{0}^{\prime }\xi_{\ell n}}, & \text{for}\;\ell \neq 0,\\ -1/\left(4\xi_{0n}^{2}\right), & \text{for}\;\ell =0. \end{array}$$ (27)

The full sum in (16) is thus

$$\begin{array}{l} \sum_{\ell \prime n\prime m\prime \neq \ell nm}\frac{{\left|A_{\ell nm\ell \prime n\prime m\prime }\right|}^{2}}{N_{\ell nm}N_{\ell \prime n\prime m\prime }k_{\ell n}^{2}\left(k_{\ell n}^{2}-k_{\ell \prime n\prime }^{2}\right)}\\ \;=\frac{4\varepsilon^{2}}{\xi_{\ell n}^{2}-\ell (\ell +1)}\sum_{\ell \prime m\prime }{\left|B_{\ell m\ell \prime m\prime }^{\left(n\right)}\right|}^{2}S_{\ell n\ell \prime }+\frac{2\varepsilon^{2}{\left|B_{\ell m00}^{\left(n\right)}\right|}^{2}}{\xi_{\ell n}^{2}\left[\xi_{\ell n}^{2}-\ell (\ell +1)\right]}, \end{array}$$ (28)

where the last term is 1/3 of the contribution from the 010-mode, which has a special normalization; 2/3 of the contribution of this term is included in the sum term.

### 3.1 Reference Eigenvalues

In order to separate out the effects of shape from the effects of volume, the perturbed eigenvalues *k*^2^ will be compared with the eigenvalues (*k*′*_ℓnm_*)^2^ of a reference sphere of the same volume *V* as the perturbed sphere. The fractional difference equals

$$\frac{k^{2}-{\left(k_{\ell nm}^{\prime }\right)}^{2}}{{\left(k_{\ell nm}^{\prime }\right)}^{2}}=\frac{{\left(ka\prime \right)}^{2}-\xi_{\ell n}^{2}}{\xi_{\ell n}^{2}},$$ (29)

where (*ka*′)^2^/*ξ*^2^*_ℓn_*, is the product of (*a*′/*a*)^2^ and the sum of 1 and the series on the right side of Eq. (16). The ratio of the volume *V* = 4π(*a*′)^3^/3 to the volume *V*_0_ = 4π*a*^3^/3 of the unperturbed sphere is

$$\begin{array}{l} {\left(\frac{a\prime }{a}\right)}^{3}=\frac{1}{4\pi }\int {\left(1-\varepsilon ℱ\right)}^{3}d\Omega \\ \;=1-3\varepsilon \langle {ℱ}_{0}\rangle +3\varepsilon^{2}\langle {ℱ}_{0}^{2}-{ℱ}_{1}\rangle +O\left(\varepsilon^{3}\right). \end{array}$$ (30)

where the triangular brackets indicate an average over solid angle. The ratio of the squared radii is

$$\left(\frac{a\prime }{a}\right)=1-2\varepsilon \langle {ℱ}_{0}\rangle +\varepsilon^{2}\left[-{\langle {ℱ}_{0}\rangle }^{2}+2\langle {ℱ}_{0}^{2}-{ℱ}_{1}\rangle \right]+O\left(\varepsilon^{3}\right).$$ (31)

The desired fractional difference is

$$\begin{array}{l} \frac{{\left(ka\prime \right)}^{2}-\xi_{\ell n}^{2}}{\xi_{\ell n}^{2}}=\frac{A_{\ell nm\ell nm}}{k_{\ell n}^{2}N_{\ell nm}}+\frac{4\varepsilon^{2}}{\xi_{\ell n}^{2}-\ell (\ell +1)}\sum_{\ell \prime m\prime }{\left|B_{\ell m\ell \prime m\prime }^{\left(n\right)}\right|}^{2}S_{\ell n\ell \prime }\\ +\frac{2\varepsilon^{2}{\left|B_{\ell m00}^{\left(n\right)}\right|}^{2}}{\xi_{\ell n}^{2}\left[\xi_{\ell n}^{2}-\ell (\ell +1)\right]}-2\varepsilon \langle {ℱ}_{0}\rangle +\varepsilon^{2}\left[-{\langle {ℱ}_{0}\rangle }^{2}+2\langle {ℱ}_{0}^{2}\rangle -2\langle {ℱ}_{1}\rangle \right]\\ -2\varepsilon \langle {ℱ}_{0}\rangle \frac{A_{\ell nm\ell nm}}{k_{\ell n}^{2}N_{\ell nm}}+O\left(\varepsilon^{3}\right), \end{array}$$ (32)

where the term coupling to the 01-mode has been made explicit.

### 3.2 Series Evaluation

Identification of the contributions to the coefficients $B_{\ell m\ell \prime m\prime }^{\left(n\right)}$ is facilitated by expressing the shape as

$${ℱ}_{0}=\sum_{\lambda \mu }c_{\lambda \mu }Y_{\lambda \mu }.$$ (33)

Equation (24) then involves linear combinations of terms of the forms

$$\langle \ell \prime m\prime \left|\lambda \mu \right|\ell m\rangle \equiv \int Y_{\ell \prime m\prime }^{*}Y_{\lambda \mu }Y_{\ell m}d\Omega .$$ (34)

and

$$\int_{=\frac{\ell \prime (\ell \prime +1)-\lambda (\lambda +1)-\ell (\ell +1)}{2}\langle \ell \prime m\prime \left|\lambda \mu \right|\ell m\rangle ,}^{Y_{\ell \prime m\prime }^{*}\left(\frac{\partial Y_{\lambda \mu }}{\partial \theta }\frac{\partial Y_{\ell m}}{\partial \theta }+\frac{1}{\sin^{2}\theta }\frac{\partial Y_{\lambda \mu }}{\partial \phi }\frac{\partial Y_{\ell m}}{\partial \phi }\right)d\Omega }$$ (35)

where Eq. (35) was obtained using the technique described in the Appendix of Ref [9]. Alternatively, Eq. (35) can be derived using the raising and lowering angular momentum operators (see, *e.g.* Ref [14]). The bracket expressions (34) vanish unless the following conditions are satisfied:

1.|ℓ′−ℓ|≤λ≤ℓ′+ℓ, (36)

2.m′=μ+m, (37)

3.ℓ′+λ+ℓmust be even. (38)

It is clear from Eqs. (2), (34), and (35) that $B_{\ell m\ell \prime m\prime }^{\left(n\right)}$ can be expressed as a linear combination of bracket ex pressions (34) with | *m*′ | ≤ *ℓ*′ and | *m* |≤ *ℓ.* Accordingly, it is possible to identify the terms that can possibly contribute to non-varnishing values of $B_{\ell m\ell \prime m\prime }^{\left(n\right)}$, by applying the following rules:

1. First look at the non-vanishing *c_λµ_* in Eq. (33).
2. For the unperturbed mode index *ℓ*, look at each expansion-coefficient index λ and find the values of *ℓ*′ satisfying the conditions of Eqs. (36) and (38).
3. Note that the coefficients α*_mµ_*_′_ in Eq. (2) are often non-zero only for *µ*′ = ±*m*. Consider the possible terms; then for each unperturbed mode index *m*, and expansion coefficient index *µ* find the value of *m*′ satisfying Eq. (37).
4. Once the possible non-vanishing coefficients $B_{\ell m\ell \prime m\prime }^{\left(n\right)}$ are identified, computation of the values of the coefficients can be carried out using symbolic algebra software.

## 4. Examples

The second order perturbations of the *ℓ* = 0 radial modes and the three-fold degenerate *ℓ* = 1 modes are worked out in this section for prolate and oblate ellipsoids. and for triaxial ellipsoids.

The eigenfunctions of the unperturbed *ℓ* = 0 modes are

$$\Phi_{0n0}=j_{0}\left(k_{0n}r\right){ϒ}_{00}.$$ (39)

The appropriate *ℓ* = 1 unperturbed eigenfunctions for any quasi-spherical resonator that has its major axes aligned with the $\hat{x}$, $\hat{y}$, and $\hat{z}$ directions are the product of *j*_1_(*k*_1_*_n_r*) and

$$\begin{array}{l} \;{ϒ}_{11}=\frac{-Y_{11}+Y_{1-1}}{\sqrt{2}}=\sqrt{\frac{3}{4\pi }}\sin\theta \cos\phi =\sqrt{\frac{3}{4\pi }}\frac{x}{r}\\ \;{ϒ}_{10}=Y_{10=}\sqrt{\frac{3}{4\pi }}\cos\theta =\sqrt{\frac{3}{4\pi }}\frac{z}{r}\\ {ϒ}_{1,-1}=\frac{-Y_{11}+Y_{1-1}}{\sqrt{2i}}=\sqrt{\frac{3}{4\pi }}\sin\theta \cos\phi =\sqrt{\frac{3}{4\pi }}\frac{y}{r}, \end{array}$$ (40)

for which the submatrix with components *A*_1_*_nm_*_,1_*_nm_*_′_ is diagonal in *mm*′.

### 4.1 Prolate Spheroid

For a spheroid of semi-major axis *a* and semi-minor axes *b* = *a*/(1 + *ε*), with *ε* > 0, the radial coordinate is

$$r=\frac{a}{\sqrt{1+(2\varepsilon +\varepsilon^{2})}\sin^{2}\theta }=a(1-\varepsilon ℱ),$$ (41)

with

$$ℱ=\sin^{2}\theta +\varepsilon \left(\frac{1}{2}\sin^{2}\theta -\frac{3}{2}\sin^{4}\theta \right)+o(\varepsilon^{2}),$$ (42)

for which

$$\langle {ℱ}_{0}\rangle =\frac{2}{3},\;\langle {ℱ}_{0}^{2}\rangle =\frac{8}{15},\;\langle {ℱ}_{1}\rangle =-\frac{7}{15}.$$ (43)

The shape function ℱ_0_ is an exact linear combination of *Y*_00_ and *Y*_20_. Accordingly. for the radial modes, the contributions to $B_{00\ell \prime m\prime }^{\left(n\right)}$ are limited to *ℓ*′= 0 and *ℓ*′= 2. For the *ℓ* = 1 modes, the contributions to $B_{1m\ell \prime m\prime }^{\left(n\right)}$ are limited to *ℓ*′=1 and *ℓ*′ = 3.

#### 4.1.1 *ℓ* = 0 Modes

The non-vanishing coefficients are

$$B_{0000}^{\left(n\right)}=\frac{2}{3}\xi_{0n}^{2},\;B_{0020}^{\left(n\right)}=\frac{2\sqrt{5}}{15}\xi_{0n}^{2}.$$ (44)

Evaluation of Eqs. (79) and (78) yields

$$S_{0n0}=\frac{1}{4\xi_{0n}^{2}},\;S_{0n2}=\frac{1}{6},$$ (45)

where the latter was obtained using the condition $j_{0}^{\prime }(\xi_{0n})=-j_{1}(\xi_{0n})=0$ and recurrence relations for the spherical Bessel functions. Evaluation of the perturbation series (32) yields

$$\frac{{\left(ka\prime \right)}^{2}-\xi_{0n}^{2}}{\xi_{0n}^{2}}=\frac{8\xi_{0n}^{2}\varepsilon^{2}}{135}+O(\varepsilon^{3}),$$ (46)

in agreement with Eq. (30) of Ref [8].

#### 4.1.2 *ℓ* = 1 Modes

Equation (22) is

$$\begin{array}{l} \frac{A_{1n01n0}}{{\left(k_{1n}\right)}^{2}N_{1n0}}=\frac{4\left(\xi_{1n}^{2}-4\right)}{5\left(\xi_{1n}^{2}-2\right)}\varepsilon -\frac{2(9\xi_{1n}^{4}-126\xi_{1n}^{2}+440)}{175{\left(\xi_{1n}^{2}-2\right)}^{2}}+O(\varepsilon^{3})\\ \frac{A_{1n\pm 1,1n\pm 1}}{{\left(k_{1n}\right)}^{2}N_{1n\pm 1}}=\frac{4\left(\xi_{1n}^{2}-3\right)}{5\left(\xi_{1n}^{2}-2\right)}\varepsilon \frac{2\left(6\xi_{1n}^{4}-259\xi_{1n}^{2}+270\right)}{175{\left(\xi_{1n}^{2}-2\right)}^{2}}\varepsilon^{2}+O(\varepsilon^{3}). \end{array}$$ (47)

The non-vanishing coefficients are

$$\begin{array}{l} B_{10,10}^{\left(n\right)}=\frac{2}{5}\left(\xi_{1n}^{2}-4\right),\\ B_{10,30}^{\left(n\right)}=-\frac{2\sqrt{21}}{35}\left(\xi_{1n}^{2}-4\right),\\ B_{1,\pm 1,1,\pm 1}^{\left(n\right)}=\frac{2}{5}(2\xi_{1n}^{2}-3),\\ B_{1,\pm 1,3,\pm 1}^{\left(n\right)}=-\frac{2\sqrt{14}}{35}(\xi_{1n}^{2}-4). \end{array}$$ (48)

From Eqs. (74) and (72), the required sums are

$$S_{1n1}=\frac{\xi_{1n}^{2}-6}{4{\left(\xi_{1n}^{2}-2\right)}^{2}},\;S_{1n3}=\frac{\xi_{1n}^{2}-5}{10\left(\xi_{1n}^{2}-4\right)},$$ (49)

the latter following from recurrence relations for the spherical Bessel functions and the condition $j_{1}{}^{\prime }(\xi_{1n})=0$. Substitution of Eqs. (43) and (47)–(49) into Eq. (32) yields

$$\frac{{\left(ka\prime \right)}^{2}-\xi_{1n}^{2}}{\xi_{1n}^{2}}=-\frac{8\left(\xi_{1n}^{2}+1\right)}{15\left(\xi_{1n}^{2}-2\right)}\varepsilon +\frac{4(54\xi_{1n}^{8}+373\xi_{1n}^{6}+495\xi_{1n}^{4}-6924\xi_{1n}^{2}+2980)}{7875\left(\xi_{1n}^{2}-2\right)}\varepsilon^{2}+O(\varepsilon^{3}),\;m=0,$$ (50)

and

$$\frac{{\left(ka\prime \right)}^{2}-\xi_{1n}^{2}}{\xi_{1n}^{2}}=\frac{4\left(\xi_{1n}^{2}+1\right)}{15\left(\xi_{1n}^{2}-2\right)}\varepsilon +\frac{2(72\xi_{1n}^{8}-961\xi_{1n}^{6}+3285\xi_{1n}^{4}-3282\xi_{1n}^{2}-2560)}{7875\left(\xi_{1n}^{2}-2\right)}\varepsilon^{2}+O(\varepsilon^{3}),\;m=\pm 1.$$ (51)

**Fig. 2:**
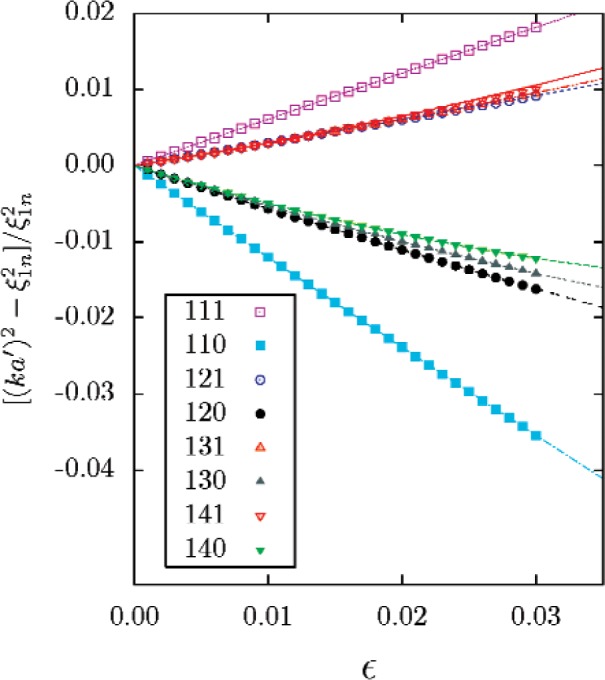
Comparison of perturbation series (lines) for prolate spheroid with exact numerical solutions (symbols).

**Fig. 3:**
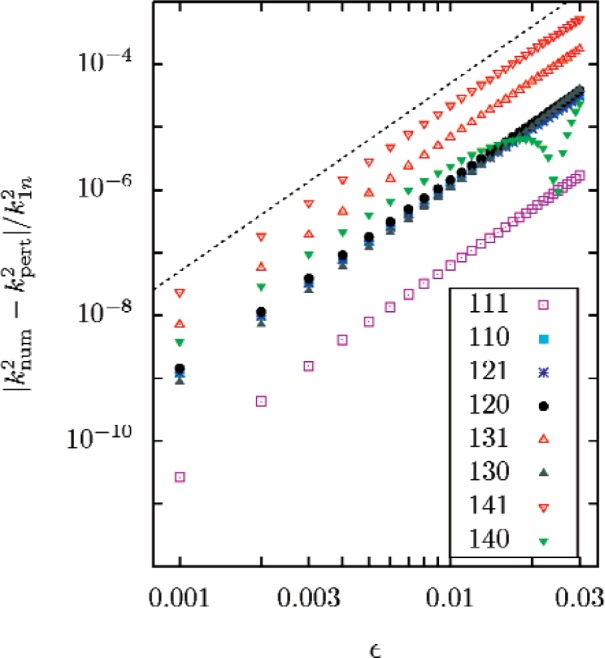
The absolute difference between the exact numerical eigenvalues $k_{\text{num}}^{2}$ and the predictions $k_{\text{pert}}^{2}$ of Eqs. (50) and (51) plotted as a fraction of *k*^2^_ln_ ≡ (*ξ*_1_*_n_*/*a*′)^2^, as functions of *ε*. The dashed line, intended as a guide to the eye, is proportional to *ε* ^3^. The plots show that the differences are approximately proportional to *ε*^3^. The numerical values exceed the perturbation values for the 111 mode over the full displayed range, and for the 140 mode for *ε* < 0.025; for all other cases the difference is negative.

The scalar Helmholtz equation separates in spheroidal coordinates, so the acoustical eigenvalues can be determined by direct numerical calculations [15]. The eigenvalues calculated numerically for a series of values of *ε* are compared with Eqs. (50) and (51) in Figs. 2 and 3.

The average eigenvalue perturbation for the 1n-triplet is, from Eqs. (50) and (51),

$${\langle \frac{{\left(ka\prime \right)}^{2}-\xi_{1n}^{2}}{\xi_{1n}^{2}}\rangle }_{1n}=\frac{8(3\xi_{1n}^{8}-14\xi_{1n}^{6}+90\xi_{1n}^{4}-243\xi_{1n}^{2}+10)}{1125{\left(\xi_{1n}^{2}-2\right)}^{3}}\varepsilon^{2}+O(\varepsilon^{3}),$$ (52)

which has no linear term, consistent with the general results derived in Ref [9].

### 4.2 Oblate Spheroid

For an oblate spheroid of semi-major axis *a* and semi-minor axes *b* = *a*/(1 + *ε*), *ε* > 0, the radial coordinate is

$$r=\frac{a}{\sqrt{1+(2\varepsilon +\varepsilon^{2})}\cos^{2}\theta }=a(1-\varepsilon ℱ),$$ (53)

with

$$ℱ=\cos^{2}\theta +\varepsilon \left(\frac{1}{2}\cos^{2}\theta -\frac{3}{2}\cos^{4}\theta \right)+O(\varepsilon^{2}),$$ (54)

for which

$$\langle {ℱ}_{0}\rangle =\frac{1}{3},\;\langle {ℱ}_{0}^{2}\rangle =\frac{1}{5},\;\langle {ℱ}_{1}\rangle =-\frac{2}{5}.$$ (55)

The perturbation calculations for the *ℓ* = 0 and *ℓ* = 1 modes parallel those for the prolate spheroid and will not be reproduced in detail here. The final expression for the eigenvalue perturbations for the radial modes is exactly the same as the result for the prolate spheroid [Eq. (46)]. For the *ℓ* = 1 modes the fractional perturbations are

$$\frac{{\left(ka\prime \right)}^{2}-\xi_{1n}^{2}}{\xi_{1n}^{2}}=\frac{8\left(\xi_{1n}^{2}+1\right)}{15\left(\xi_{1n}^{2}-2\right)}\varepsilon +\frac{4(54\xi_{1n}^{8}-677\xi_{1n}^{6}+3645\xi_{1n}^{4}-6924\xi_{1n}^{2}-1220)}{7875{\left(\xi_{1n}^{2}-2\right)}^{3}}\varepsilon^{2}+O(\varepsilon^{3}),\;m=0,$$ (56)

$$\frac{{\left(ka\prime \right)}^{2}-\xi_{1n}^{2}}{\xi_{1n}^{2}}=-\frac{4\left(\xi_{1n}^{2}+1\right)}{15\left(\xi_{1n}^{2}-2\right)}\varepsilon +\frac{2(72\xi_{1n}^{8}+89\xi_{1n}^{6}+135\xi_{1n}^{4}-3282\xi_{1n}^{2}+1640)}{7875\left(\xi_{1n}^{2}-2\right)}\varepsilon^{2}+O(\varepsilon^{3}),\;m=\pm 1.$$ (57)

Exact solutions for the oblate spheroid [16] were calculated and compared with Eqs. (56) and (57). The agreement, like the corresponding agreement for the prolate spheroid, is very good. The plots resemble Figs. 2 and 3.

The mode average is exactly the same as Eq. (52) for the prolate spheroid.

### 4.3 Triaxial Ellipsoid

The surface of the triaxial ellipsoid defined by

$$\frac{x^{2}}{{\left(1+\varepsilon_{2}\right)}^{2}}+y^{2}+\frac{z^{2}}{{\left(1+\varepsilon_{1}\right)}^{2}}=\frac{a^{2}}{{\left(1+\varepsilon_{1}\right)}^{2}{\left(1+\varepsilon_{2}\right)}^{2}}$$ (58)

can be expressed in the form of Eq. (3) with

$$\varepsilon {ℱ}_{0}=\varepsilon_{1}\sin^{2}\theta +\varepsilon_{2}(\cos^{2}\theta +\sin^{2}\theta \sin^{2}\phi )$$ (59)

and

$$\begin{array}{l} \varepsilon^{2}{ℱ}_{1}=\varepsilon_{1}^{2}\left(\frac{3}{2}\cos^{2}\theta -1\right)\sin^{2}\theta \\ \;+\varepsilon_{1}\varepsilon_{2}\left(-\sin^{2}\phi -3\cos^{2}\theta \cos^{2}\phi \right)\sin^{2}\theta \\ \;+\varepsilon_{2}^{2}\left(-1+\frac{5}{2}\sin^{2}\theta \cos^{2}\phi -\frac{3}{2}\sin^{4}\theta \cos^{4}\phi \right) \end{array}$$ (60)

The shape ℱ_0_ is an exact linear combination of *Y*_00_, *Y*_20_, and *Y*_2,±2_. The non-vanishing values of $B_{\ell m\ell \prime m\prime }^{\left(n\right)}$ are accordingly limited to the same values of ℓ′ as for the spheroids.

#### 4.3.1 *ℓ* = 0 Modes

The non-vanishing coefficients are

$$\begin{array}{l} \varepsilon B_{0000}^{\left(n\right)}=\frac{2}{3}\xi_{0n}^{2}(\varepsilon_{1}+\varepsilon_{2}),\\ \varepsilon B_{0000}^{\left(n\right)}=-\frac{\sqrt{5}}{15}\xi_{0n}^{2}(2\varepsilon_{1}-\varepsilon_{2}). \end{array}$$ (61)

Evaluation of the perturbation series (32) yields

$$\frac{{\left(ka\prime \right)}^{2}-\xi_{0n}^{2}}{\xi_{0n}^{2}}=\frac{8\xi_{0n}^{2}}{135}(\varepsilon_{1}^{2}-\varepsilon_{1}\varepsilon_{2}+\varepsilon_{2}^{2})+O(\varepsilon^{3}).$$ (62)

**Fig. 4:**
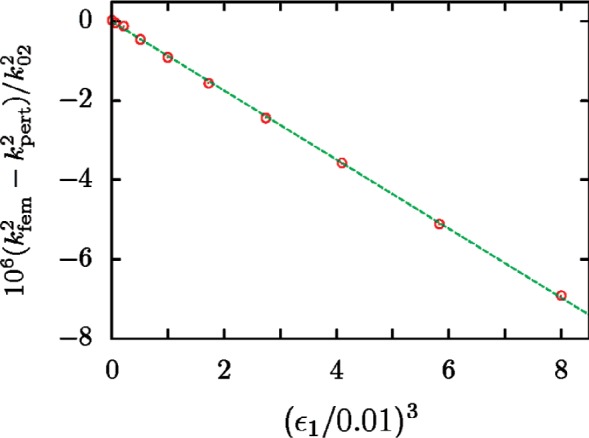
The differences between the values of $k_{\text{fem}}^{2}$ determined with the finite-element method for the 02 radial mode and the predictions $k_{\text{pert}}^{2}$ of Eq. (62) for an ellipsoid with *ε*_2_ = *ε*_1_/2. The line proportional to $\varepsilon_{1}^{3}$ was fit to the plotted points.

The correctness of this result was checked by calculating the radial-mode eigenvalues of a triaxial ellipsoid using a finite-element method. The parameters *ε*_1_ and *ε*_2_ were varied, with the ratio held constant at *ε*_1_/*ε*_2_ = 2. Figure 4 shows that the difference between the finite-element results and Eq. (62) is cubic in *ε* ^3^.

#### 4.3.2 *ℓ* = 1 Modes

The non-vanishing coefficients $B_{\ell m\ell \prime m\prime }^{\left(n\right)}$ are, with *ξ* =*ξ*_1_*_n_* for brevity,

$$\begin{array}{l} B_{1,-1,1,-1}^{\left(n\right)}=\frac{2}{5}(2\xi^{2}-3)(\varepsilon_{1}+\varepsilon_{2}),\\ B_{1,0,1,0}^{\left(n\right)}=\frac{2}{5}\left[(\xi^{2}-4)\varepsilon_{1}+{\left(2\varepsilon^{2}-3\right)}_{\varepsilon_{2}}\right],\\ B_{1,1,1,1}^{\left(n\right)}=\frac{2}{5}\left[(2\xi^{2}-3)\varepsilon_{1}+{\left(\xi^{2}-4\right)}_{\varepsilon_{2}}\right],\\ B_{1,-1,3,-3}^{\left(n\right)}=-\sqrt{\frac{3}{70}}(\xi^{2}-4)\varepsilon_{2},\\ B_{1,-1,3,-1}^{\left(n\right)}=-\frac{\sqrt{14}}{70}(\xi^{2}-4)(4\varepsilon_{1}-\varepsilon_{2}),\\ B_{1,0,3,0}^{\left(n\right)}=-\frac{\sqrt{21}}{35}(\xi^{2}-4)(2\varepsilon_{1}-\varepsilon_{2}),\\ B_{1,1,3,1}^{\left(n\right)}=-\frac{\sqrt{14}}{70}(\xi^{2}-4)(4\varepsilon_{1}-3\varepsilon_{2}),\\ B_{1,0,3,2}^{\left(n\right)}=-\frac{1}{\sqrt{35}}(\xi^{2}-4)\varepsilon_{2},\\ B_{1,1,3,3}^{\left(n\right)}=-\sqrt{\frac{3}{70}}(\xi^{2}-4)\varepsilon_{2}. \end{array}$$ (63)

The fractional perturbations for the 1*nm* modes have the form

$$\begin{array}{l} \frac{{\left(ka\prime \right)}^{2}-\xi_{1n}^{2}}{\xi_{1n}^{2}}=-\frac{4\left(\xi^{2}+1\right)(p_{1m}\varepsilon_{1}+p_{2m}\varepsilon_{2})}{15\left(\xi^{2}-2\right)}\\ \;+\frac{p_{11m}\varepsilon_{1}^{2}+p_{12m}\varepsilon_{1}\varepsilon_{2}+p_{22m}\varepsilon_{2}^{2}}{7875{\left(\xi^{2}-2\right)}^{3}}, \end{array}$$ (64)

where

$$\begin{array}{l} p_{1,1}=p_{1,-1}=p_{2,0}=p_{2,-1}=1,\\ p_{1,0}=p_{2,1}=-2,\\ p_{11,1}=2(72\xi^{8}-961\xi^{6}+3285\xi^{4}-3282\xi^{2}-2560),\\ p_{12,1}=-8(27\xi^{8}-76\xi^{6}+1035\xi^{4}-3462\xi^{2}-440),\\ p_{22,1}=4(54\xi^{8}+373\xi^{6}+495\xi^{4}-6924\xi^{2}-2980),\\ p_{11,0}=p_{22,1,}\\ p_{12,0}=p_{12,1,}\\ p_{22,0}=p_{11,1,}\\ p_{11,-1}=p_{11,1,}\\ p_{12,-1}=-8(9\xi^{8}-142\xi^{6}-180\xi^{4}+1821\xi^{2}-670),\\ p_{22,-1}=p_{11,1}. \end{array}$$ (65)

These equations have the appropriate limits when either *ε*_l_ or *ε*_2_ is zero. The mode average is

$$\begin{array}{l} {\langle \frac{{\left(ka\prime \right)}^{2}-\xi_{1n}^{2}}{\xi_{1n}^{2}}\rangle }_{1n}=\frac{8(3\xi_{1n}^{8}-14\xi_{1n}^{6}+90\xi_{1n}^{4}-243\xi_{1n}^{2}+10)}{1125{\left(\xi_{1n}^{2}-2\right)}^{3}}\\ \;\left(\varepsilon_{1}^{2}-\varepsilon_{1}\varepsilon_{2}+\varepsilon_{2}^{2}\right)+O\left(\varepsilon^{3}\right), \end{array}$$ (66)

**Fig. 5:**
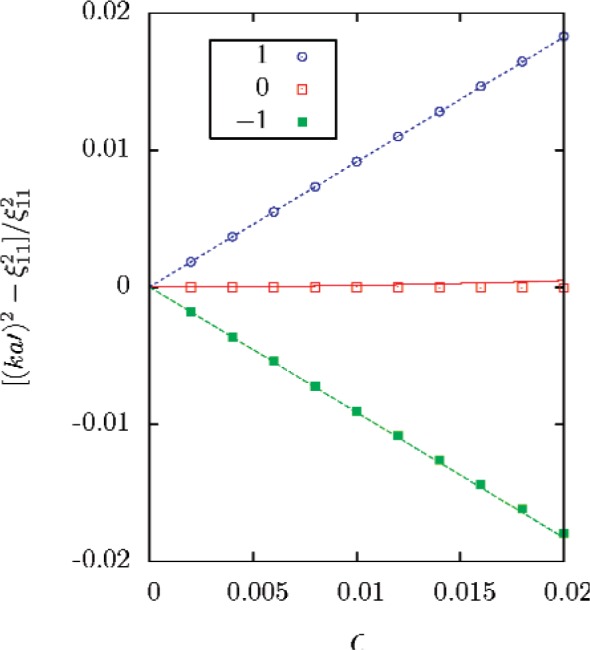
Fractional eigenvalue perturbations for the 11 *m* modes: the points are numerical results determined with the finite-element method, the lines represent Eqs. (64)–(65) for an ellipsoid with *ε*_2_ = *ε*_1_/2. The key identifies the lines by the value of the index *m*.

The correctness of Eqs. (64) and (65) was tested by calculating the modes of a triaxial ellipsoid using the finite-element method. The parameters *ε*_l_ and *ε*_2_ were varied, with the ratio held constant at *ε*_l_/*ε*_2_ = 2. This choice corresponds to a uniform splitting of the triplet (the case considered in Ref [10]) as shown in Fig. 5.

**Fig. 6:**
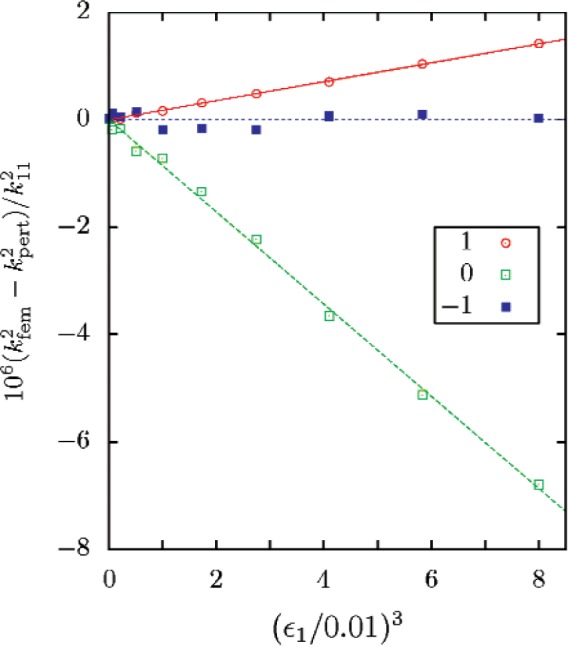
The differences between the values of $k_{\text{fem}}^{2}$ determined with the finite-element method for the 11 *m* modes and the predictions $k_{\text{pert}}^{2}$ of Eqs. (64)–(65) for an ellipsoid with *ε*_2_ = *ε*_1_/2. The lines proportional to $\varepsilon_{1}^{3}$ were fit to the plotted points. The key identifies the lines by the value of the index *m*.

Figures 5 and 6 show the close agreement of the eigenvalues determined with the finite-element method and Eqs. (64) (65).

## 5. Concluding Remarks

The formalism derived in this article can be applied, in principle, to arbitrary quasi-spherical resonators whose shape can be represented by Eq. (3). Section 3.2 lists the general principles that determine the possible contributions to the general series (32). Once the possible terms are identified, the use of symbolic algebra software can be used to calculate the terms. For increasingly complex shapes, this process should be programmed so as to minimize human error.

## 6. Appendix. Evaluation of Infinite Sums

The sum $S_{\ell n\ell^{\prime }}$ defined by Eq. (25) can be evaluated explicitly. For the case *ℓ*′ ≠ 0, consider the contour integral

$$I=\int_{C}\frac{1}{\xi^{2}-\xi_{\ell n}^{2}}\frac{\xi^{2}}{\xi^{2}-\ell \prime (\ell \prime +1)}\frac{j_{\ell \prime }^{\prime \prime }(\xi )}{j_{\ell \prime }^{\prime }(\xi )}d\xi ,$$ (67)

where *C* is a rectangular contour with corners at (± *x_N_*, ±*y*_0_), with *y*_0_ > 0 and *x_N_* sufficiently large that *N* zeros of $j_{{\;}^{\prime }\ell }^{\prime }(\xi )$ lie along the positive real axis within *C*. The integrand is bounded on *C*, so the integral approaches zero as *N* → ∞; the sum of all residues within the contour is also zero in this limit.

The integrand has poles at ±*ξ_ℓn_*, $\sqrt{\ell \prime (\ell \prime +1)}$, and ±*ξ*_ℓ′_*_ν_* for *ν* =1,2,…*N*. When *ℓ′* ≠ *ℓ* the poles are all first order, with residues

$$ℛ(\pm \xi_{\ell n})=-\frac{1}{\xi_{\ell n}^{2}-\ell \prime (\ell \prime +1)}-\frac{j_{\ell \prime }(\xi_{\ell n})}{2\xi_{\ell n}j_{\ell \prime }^{\prime }(\xi_{\ell n})},$$ (68)

and

$$ℛ\left(\pm \sqrt{\ell \prime (\ell \prime +1)}\right)=-\frac{1}{\xi_{\ell n}^{2}-\ell \prime (\ell \prime +1)},$$ (69)

$$ℛ(\pm \xi_{\ell \prime \nu })=-\frac{1}{\xi_{\ell n}^{2}-\xi_{\ell \prime \nu }^{2}}\frac{\xi_{\ell \prime \nu }^{2}}{\xi_{\ell \prime \nu }^{2}-\ell \prime (\ell \prime +1)}.$$ (70)

The sum of all residues ℛ(±*ξ_ℓ′µ_*) is $-2S_{\ell n\ell^{\prime }}$, so the condition that the sum of all residues is zero implies

$$-S_{\ell n\ell \prime }+ℛ(\xi_{\ell n})+ℛ\left(\sqrt{\ell \prime (\ell \prime +1)}\right)=0,$$ (71)

or

$$S_{\ell n\ell \prime }=-\frac{j_{\ell \prime }(\xi_{\ell n})}{2\xi_{\ell n}j_{\ell \prime }^{\prime }(\xi_{\ell n})},\;\ell \prime \neq \ell .$$ (72)

When *ℓ'* = *ℓ* the pole at *ξ_ℓn_* is second order, with residue

$$ℛ(\pm \xi_{\ell n})=-\frac{3\xi_{\ell n}^{2}-\ell (\ell +1)}{4{\left[\xi_{\ell n}^{2}-\ell (\ell +1)\right]}^{2}}.$$ (73)

and the sum of the series is

$$S_{\ell n\ell }=-\frac{\xi_{\ell n}^{2}-3\ell (\ell +1)}{4{\left[\xi_{\ln}^{2}-\ell (\ell +1)\right]}^{2}}.$$ (74)

Next consider the case *ℓ*′ = 0. The second factor in the integrand of the contour integral is then unity; the residues are

$$ℛ(\pm \xi_{\ell n})=\frac{1}{\xi_{\ell n}^{2}}-\frac{j_{0}(\xi_{\ell n})}{2\xi_{\ell n}^{2}j_{0}^{\prime }(\xi_{\ell n})},\;\ell \neq 0,$$ (75)

and

$$ℛ(\pm \xi_{\ell \prime \nu })=-\frac{1}{\xi_{\ell n}^{2}-\xi_{\ell \prime \nu }^{2}}.$$ (76)

There is a single pole at *ξ*_01_ = 0, so the sum of all residues is

$$\sum_{\nu =-\infty }^{\infty }ℛ(\pm \xi_{\ell \prime \nu })+2ℛ(\pm \xi_{\ell n})=\left[-2S_{\ell n0}+\frac{1}{\xi_{\ell n}^{2}}\right]-\left[\frac{2}{\xi_{\ell n}^{2}}+\frac{j_{0}(\xi_{\ell n})}{\xi_{\ell n}j_{0}^{\prime }(\xi_{\ell n})}\right]=0,$$ (77)

which implies

$$S_{\ell n0}=-\frac{1}{2\xi_{\ell n}^{2}}+\frac{j_{0}(\xi_{\ell n})}{2\xi_{\ell n}j_{0}^{\prime }(\xi_{\ell n})}.$$ (78)

When ℓ = 0 the (distinct) poles at ±*ξ*_0_*_n_* are second order; the residues are $-3/(4\xi_{0n}^{2})$, so the sum is

$$S_{0n0}=-\frac{1}{4\xi_{0n}^{2}}.$$ (79)
